# States of epistemic curiosity interfere with memory for incidental scholastic facts

**DOI:** 10.1038/s41539-024-00234-w

**Published:** 2024-03-18

**Authors:** Nicole E. Keller, Carola Salvi, Emily K. Leiker, Matthias J. Gruber, Joseph E. Dunsmoor

**Affiliations:** 1 Institute for Neuroscience, University of Texas at Austin, Austin, TX USA; 2 Department of Psychology and Social Sciences, John Cabot University, Rome, Italy; 3 Department of Psychiatry, School of Medicine, University of Pittsburgh, Pittsburgh, PA USA; 4 Cardiff University Brain Research Imaging Centre (CUBRIC), School of Psychology, Cardiff University, Maindy Road, Cardiff, CF24 4HQ UK; 5 Department of Neuroscience, University of Texas at Austin, Austin, TX USA; 6 Department of Psychiatry and Behavioral Sciences, Dell Medical School, University of Texas at Austin, Austin, TX USA

**Keywords:** Human behaviour, Problem solving

## Abstract

Curiosity can be a powerful motivator to learn and retain new information. Evidence shows that high states of curiosity elicited by a specific source (i.e., a trivia question) can promote memory for incidental stimuli (non-target) presented close in time. The spreading effect of curiosity states on memory for other information has potential for educational applications. Specifically, it could provide techniques to improve learning for information that did not spark a sense of curiosity on its own. Here, we investigated how high states of curiosity induced through trivia questions affect memory performance for unrelated scholastic facts (e.g., scientific, English, or historical facts) presented in close temporal proximity to the trivia question. Across three task versions, participants viewed trivia questions closely followed in time by a scholastic fact unrelated to the trivia question, either just prior to or immediately following the answer to the trivia question. Participants then completed a surprise multiple-choice memory test (akin to a pop quiz) for the scholastic material. In all three task versions, memory performance was poorer for scholastic facts presented after trivia questions that had elicited high versus low levels of curiosity. These results contradict previous findings showing curiosity-enhanced memory for incidentally presented visual stimuli and suggest that target information that generates a high-curiosity state interferes with encoding complex and unrelated scholastic facts presented close in time.

## Introduction

Curiosity can produce a strong motivation to acquire new information^1–3^. There is increasing evidence that seeking to satisfy curiosity drives the desire to learn new information and helps enhance that information in long-term memory^4^. A state of curiosity may enhance retention through heightened attention during memory formation and prioritize consolidation of material that piqued high interest. Research on the neurocognitive mechanisms underlying curiosity, therefore, provides direct implications for education; specifically, could strategies that promote curiosity in the classroom lead to better learning and retention of educational material^5^? An intriguing finding from recent research reveals that curiosity-enhanced memory has the potential to spread to incidental material presented during high states of curiosity^6–9^ (but also see ref. ^10^). In other words, long-term memory is enhanced for seemingly random stimuli that just happened to be presented while participants learn about information they are curious about. Harnessing curiosity states could provide a route to help improve learning and retention for information that does not spark a sense of curiosity and is therefore more challenging to learn and retain. The present study investigated whether high states of curiosity have a carryover effect for incidental presentations of unrelated scholastic facts appearing close in time.

The spread of curiosity-enhanced memory to incidental stimuli suggests a phasic neurocognitive state that can generalize beyond the goal-relevant material that generated that state. Neuroimaging evidence reveals curiosity-enhanced memory is driven by activity in the dopaminergic system modulating activity in the hippocampus^11,12^, similar in many respects to the neurocircuitry involved in reward and novelty-enhanced memory^13,14^. Prior research shows that stimuli presented in the anticipatory period between a trivia question and its associated answer also benefit from this neurocircuitry, leading to better memory for these incidentally presented items. Indeed, Gruber et al.^11^ found that individual differences in the midbrain and hippocampal activity elicited by trivia questions correlated with memory enhancements for incidental stimuli.

Notably, the incidental stimuli used in prior research have included visual stimuli, often pictures of faces e.g., refs. ^8,11^. Whether curiosity enhances memory for more complex incidental information is unknown. This question is important for translation to educational applications, as the type of information one would hope to benefit from curiosity-based interventions would be more complex than recognition of simple visual stimuli. One possibility is that seeking to resolve the uncertainty of the target information (e.g., a trivia question) captures different types of incidental information in memory, irrespective of the complexity. In this case, we predict better performance on a test of scholastic facts presented in the anticipatory period when curiosity is piqued versus when curiosity is low. However, an alternative hypothesis is that stimulus complexity is a boundary condition for this generalized curiosity-enhanced memory effect, mitigating the enhancement of non-target information. For example, according to an arousal-biased competition model of memory allocation^15^, the goal state to resolve the uncertainty of the trivia question would interfere with the encoding of unrelated incidental information. Compared to incidental visual stimuli used in prior trivia paradigms e.g., refs. ^8,11^, interference may be particularly pronounced for scholastic information, as it is likely to compete for similar processing resources as the trivia material. Given they are of a similar epistemic nature, a trivia question may interfere with the processing of unrelated scholastic information. That is, both the scholastic information and the trivia question concern knowledge that participants are voluntarily motivated to attend to without any external reinforcement to encode the information; but a more intriguing trivia question could overshadow unrelated knowledge presented close in time. In this case, we should predict worse performance on a test of scholastic facts presented while participants were highly curious about an unrelated trivia fact.

The present study aimed to investigate whether curiosity improves or diminishes test performance for unrelated scholastic facts presented in temporal proximity to high states of curiosity. Whether curiosity enhances or diminishes retention of unrelated scholastic information is vital to resolve, as it may have practical significance in classroom settings^16,17^. For example, piquing curiosity might be a strategy for bolstering retention of unrelated material that is less intrinsically interesting and more difficult to retain in memory^18^; alternatively, piquing strong curiosity might interfere with memory formation for other material presented too close in time to the material that elicited curiosity. In essence, curiosity could overshadow the encoding of less stimulating information presented in close temporal proximity. These competing hypotheses could have implications for how curiosity is leveraged as a cognitive adjunct in classroom settings.

In a between-subjects design using three slightly modified versions of the same task, we adapted the trivia paradigm from Gruber et al.^11^ and presented an incidental scholastic fact at the moment between the trivia question and the answer, or after the answer when curiosity was satisfied. Scholastic facts spanned topics of science, history and English, and were unrelated to the trivia questions (e.g., trivia about celebrities). Following the trivia paradigm (encoding), participants underwent a surprise multiple choice test (retrieval) for the scholastic material, analogous to a pop-quiz.

## Results

### Main task

**Fig. 1:**
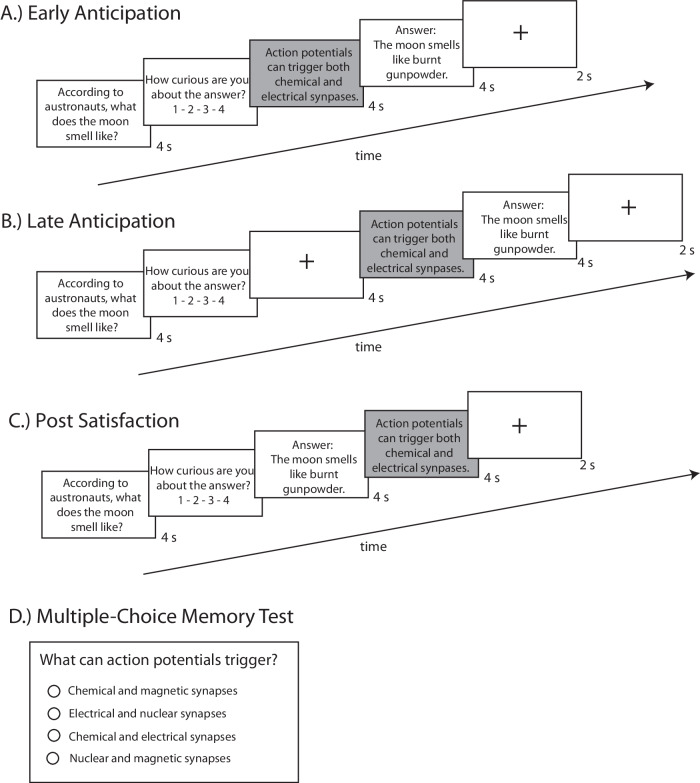
Study design. **A** Early Anticipation Version. For each trial, a selected trivia question was presented and participants had to rate their level of curiosity. Immediately following this rating, participants were presented with a scholastic fact, unrelated to the trivia question. Following the presentation of a scholastic fact, participants would receive the answer to the trivia question. **B** Late Anticipation. This version was the same as the Early Anticipation design, with the critical exception of the presentation of a 4-s crosshair between the trivia rating and scholastic fact. **C** Post Satisfaction. In this version, participants were initially presented with a trivia question immediately followed by the answer. Unlike the first two versions, the scholastic fact in this version was presented after the trivia answer. **D** Following all study phase versions, participants completed a surprise multiple-choice memory test on the scholastic facts.

The design was adapted from (Gruber et al.^11^) and was programmed and distributed online using Qualtrics (Qualtrics, Provo, UT). As shown in Fig. 1, we tested three versions of the task that varied the appearance of a scholastic fact in relation to the trivia question and answer. In two versions of the task, in line with Gruber et al.^11^, we examined whether an anticipatory period during a state of curiosity could enhance memory for incidental scholastic facts. In the first version (Early Anticipation), the scholastic fact appeared immediately after participants rated their curiosity for the preceding trivia question. In the second version (Late Anticipation), there was a delay of 4 s following the curiosity rating before the scholastic fact appeared, intended to generate a slightly more prolonged state of curiosity before the answer to the trivia question was presented. In the third version of the task (Post Satisfaction), the scholastic fact appeared immediately following the answer to the trivia question, allowing us to examine whether the satisfaction generated by receiving the answer to a trivia question affected memory for the incidental scholastic fact. Participants were informed that they would see some facts that may not seem related to the trivia questions, but that it was still necessary to pay close attention to them. Participants who completed one of the task versions were prevented from participating in the other two task versions, and participant assignment was quasi-random.

### Pre-determined high vs. low curiosity ratings

In our main task, we used trivia questions pilot-rated by a separate group of participants. These pilot-rated trivia questions were divided into low- and high-curiosity categories (see methods for more details). To confirm participants in all three versions of the main task were rating pre-determined low vs. high curiosity trivia questions (rated 1–4) accordingly (i.e., giving the pre-determined low curiosity category of trivia facts a significantly lower rating than the pre-determined high curiosity trivia facts), we conducted an ANOVA of average curiosity ratings with task version (early anticipation, late anticipation, and post satisfaction) as a between-subjects factor and pre-determined curiosity rating (low vs. high) as a within-subjects factor. This analysis revealed a main effect of the pre-determined curiosity category (F_(1, 236)_ = 469.80, *p* < 0.001, η^2^_G_ = 0.322), no main effect of task version (*p* = 0.299), and no significant interaction (*p* = 0.055). As expected, post hoc paired *t-*tests revealed significantly higher ratings for the pre-determined high curiosity category than the low curiosity category, in all task versions (all *ps* < 0.001). Further, pre-determined category ratings were not different between task versions for either the high curiosity category (early anticipation vs late anticipation 1.2: *p* = 0.667, early anticipation vs post satisfaction: *p* = 0.216, late anticipation vs. post satisfaction: *p* = 0.182), nor the low curiosity category (early anticipation vs late anticipation: *p* = 0.057, early anticipation vs post satisfaction: *p* = 0.330, late anticipation vs. post satisfaction: *p* = 0.280). Thus, we confirmed that trivia questions from our pre-determined curiosity categories were rated as expected in the main task, and that there were no differences amongst task versions (for mean and SEM see Table 1).

**Table 1:** Mean ± SEM curiosity ratings for pre-determined high vs. low curiosity trivia categories

Study version	Pre-determined high curiosity	Pre-determined low curiosity
Early Anticipation	3.244 ± 0.051	2.163 ± 0.070
Late Anticipation	3.283 ± 0.078	2.410 ± 0.117
Post Satisfaction	3.142 ± 0.064	2.264 ± 0.076

### Pre-determined high curiosity trivia questions are associated with lower memory of incidental scholastic material

**Fig. 2:**
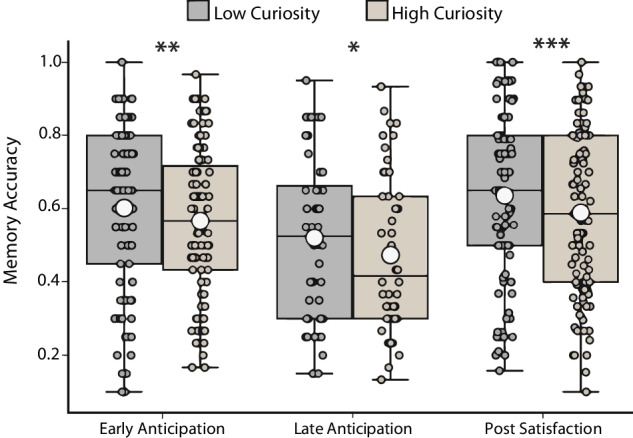
Memory for scholastic facts paired to pre-determined high vs. low curiosity trivia questions. In all task versions, memory for scholastic facts was significantly lower when paired with a high vs. a low curiosity trivia question. This box plot depicts the median (center line), upper and lower quartiles (box limits), maximum to the upper quartile (upper whisker), and lower quartile to the minimum (lower whisker). White circles depict memory accuracy means. Individual data points are depicted, and there were no outliers. ***p < 0.001, **p < 0.01, *p < 0.05.

Next, we explored the effects of pre-determined low vs. high curiosity states on memory for incidentally encoded scholastic facts. An ANOVA of scholastic fact memory with task version as a between-subjects factor, and pre-determined curiosity category as a within-subjects factor (low vs. high), revealed a main effect of task version (F_(2, 236)_ = 4.80, *p* = 0.009, η^2^_G_ = 0.036) and a main effect of curiosity category (F_(1, 236)_ = 25.05, *p* < 0.001, η^2^_G_ = 0.009), but no interaction (*p* = 0.788). Post hoc paired *t*-tests revealed that within all versions of the task, memory was significantly lower for scholastic facts in the high as compared to the low curiosity condition (Early Anticipation: t_(94)_ = −2.940, *p* = 0.004, 95% CI [−0.060, −0.012]; Late Anticipation: t_(47)_ = −2.478, *p* = 0.017, 95% CI [−0.086, −0.009]; Post Satisfaction: t_(95)_ = −3.467, *p* < 0.001, 95% CI [−0.074, −0.020]) (Fig. 2). Thus, higher states of curiosity generated by a trivia question were associated with worse memory for unrelated and incidentally presented scholastic facts, regardless of the temporal proximity of the curiosity state.

### High curiosity states associated with poorer memory for incidental scholastic facts

**Fig. 3:**
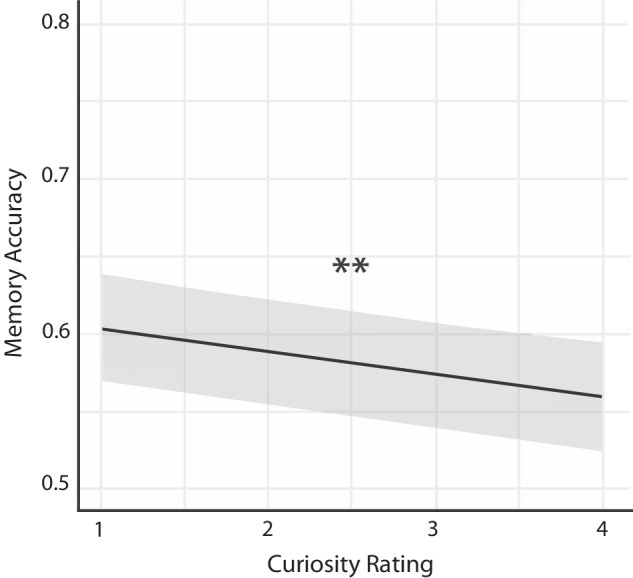
Predicted probabilities of memory accuracy for scholastic facts. A generalized mixed model with all task versions predicted a significant decline in memory for scholastic facts paired to trivia questions with higher curiosity ratings. The error bar represents standard error.

We then tested the effect of participants’ individual trial-level curiosity ratings (as opposed to pre-determined ratings) on memory for the scholastic fact accompanying that trial. As we observed the same effect in all task versions for the pre-determined ratings, we constructed a generalized mixed model with all task versions to explore whether individual higher states of curiosity were associated with lower memory. Specifically, we included fixed effects of task version (Early Anticipation, Late Anticipation, Post Satisfaction), trial level curiosity ratings (1–4), trial number, and mean curiosity rating per participant (to account for between-subjects variance in average curiosity). In accordance with the findings using pre-determined levels of curiosity, this model revealed a significant effect of curiosity ratings, so that lower ratings were associated with higher memory performance (*OR* = 0.93, 95% CI [0.89, 0.98], *z*_wald_ = −2.90, *p* = 0.004) (Fig. 3). There was also a significant effect of task version, between Early Anticipation vs. Late Anticipation (*OR* = 0.68, 95% CI [0.48, 0.97], *z*_wald_ = −2.12, *p* = 0.034) but no effect of Early Anticipation vs. Post Satisfaction (*OR* = 1.18, 95% CI [0.89, 1.57], *z*_wald_ = 1.13, *p* = 0.258). Notably, there was no effect of trial number (*OR* = 0.97, 95% CI [0.93, 1.01], *z*_wald_ = −1.39, *p* = 0.166) nor average curiosity rating per participant (*OR* = 0.95, 95% CI [0.83, 1.08], *z*_wald_ = −0.76, *p* = 0.448), indicating that neither trial order nor average participant level curiosity rating affected memory accuracy. Overall, these results further confirm that higher curiosity states interfered with memory for scholastic facts, as scholastic facts paired with lower curiosity-rated trivia questions were remembered better than those paired with higher curiosity-rated trivia questions.

## Discussion

Optimal strategies to enhance engagement with scholastic material to bolster learning, retention, and comprehension has remained a major topic of education research. The relationship between curiosity and learning has been of particular empirical interest, as information that piques curiosity tends to be remembered better^17^. Although whether curiosity, per se, is the optimal route to enhance engagement in classroom settings is not resolved. There is intuitive appeal to the idea that enhancing engagement through curiosity enhances learning of that material. Recent intriguing findings show that states of curiosity carry forward to enhance memory for unrelated visual information that is presented close in time, on the order of seconds^6–9^. Here, we tested the hypothesis that this carry-over effect extends to more complex scholastic information, such that information presented after trivia questions that piqued curiosity would selectively improve test performance for this information. However, our study results across three task versions suggest the opposite. Specifically, test performance was selectively impaired for scholastic information presented after high-curiosity trivia questions.

One framework to interpret the present results concerns competition between the trivia question and target material. Specifically, the goal-state to resolve uncertainty of high curiosity trivia questions may have overshadowed processing the target material presented. Similar competition effects have been reported in studies of emotional memory, whereby emotional stimuli have divergent modulatory effects on incidental stimuli presented in close temporal proximity^15,19–21^. In some cases, an emotional stimulus enhances memory for unrelated stimuli; whereas in other cases it impairs memory. Mather and colleagues proposed that arousal will enhance memory when there is need for selective attention toward the target information, such that the information is of high priority or matches the goal-state of the individual^15^. Arousal might also lead to enhanced memory for bottom-up information that “pops out,” thus amplifying the salience of otherwise neutral information. This effect might in part explain research showing carryover effects of curiosity for incidental visual information (i.e., faces) that contrast with the nature of the trivia question. In this framework, relevant or contrasting neutral information presented around the time of phasic increases in arousal will gain priority and will be preferentially remembered^15^. Alternatively, arousal can narrow the focus of attention toward stimuli that are irrelevant to the source of arousal^22^. Support of this effect is found through the focus on the arousing details of a scene at the expense of memory for the neutral background or contextual details. Although in this experiment the trivia question and scholastic fact were separated in time, high curiosity trivia might have narrowed attention and interfered with encoding the scholastic information. The fact that both events were of a similar epistemic nature likely blunted bottom-up “pop-out” processing in the present study as compared to prior studies. The overlap between arousal generally, and curiosity more specifically, merits further investigation.

The question remains: could inducing states of curiosity be used to improve learning for other information that does not pique a sense of curiosity on its own? One of the boundary conditions may simply be the timing between the two events. Here, we used a trial-by-trial design in keeping with a typical laboratory-based memory encoding paradigm. The target memoranda were presented immediately or shortly following the question or the answer, on a trial-by-trial basis. However, in real-world applications, curiosity would likely be a more sustained (tonic) state that could be induced over the course of several minutes. For example, students might engage with a topic that is of high interest for several minutes prior to learning about material that is of less interest and therefore more challenging to learn. (Note: the distinction between “interest” and “curiosity” is still a matter of debate^23^, though here we used the terms interchangeably). This could allow a tonic state of curiosity to build up over time. Waiting some amount of time to pass after a tonic curiosity state might then prevent overshadowing of the target information, whilst retaining the neuromodulatory benefits of the state. Whether such a tonic state of curiosity is neurobiologically supported is less clear, given that functional neuroimaging of curiosity has used the type of trial-by-trial alterations in high versus low curiosity states used here. But there is support for the notion of fast and slow timescales of dopaminergic activity throughout the midbrain, hippocampus, and prefrontal cortex in human fMRI^24^ that accords with neurobiological recordings of dopaminergic neurons^25,26^. Importantly, phasic bursts versus sustained tonic release of dopamine has different effects on learning, memory, and behavior^13,27^.

Another avenue that should be addressed in future investigations is the role of consolidation^28,29^ on curiosity-enhanced memory for incidental information. Here, memory was assessed in a surprise multiple-choice “pop quiz” immediately following encoding. However, consolidation has selective effects on arousal-modulated memory^29^ that might be relevant to curiosity as well. One exciting avenue for future research concerns a model of memory capture for weak memories formed within a time window surrounding a novel or salient event, known as behavioral tagging or synaptic tag-and-capture^30–32^. In this model, a strong experience will increase the likelihood that a weak memory will be consolidated to form a durable long-term memory if the weak memory is formed *before* or *after* the strong event. If a high state of curiosity can be framed as a strong learning event, then one prediction is that a state of curiosity works retroactively to bolster retention of information learned prior to the state of curiosity. Notably, this model would propose that the effect would be observed after a state of consolidation. Curiosity may be one means to induce novelty. Interestingly, the idea of incorporating novelty in a behavioral tagging framework already has empirical support in classroom environments^33,34^. It is important to note that some studies using the trivia paradigm that have found positive effects of enhanced memory for incidental visual stimuli used a 24-h memory test delay^8,11^. Stare and colleagues tested the effects of immediate versus delayed testing and found spillover effects across both conditions^7^. The importance of the consolidation period for curiosity carry-over effects is at this point unclear.

There are limitations to the present study that should be accounted for in future studies in this area. First, the study was conducted online during the COVID-19 pandemic. Conducting research during this anxiety-provoking global event could affect results generally. However, at least two studies suggest that curiosity—even accompanied by increasing anxiety levels during the COVID-19 pandemic—had an effect on information seeking during the pandemic^35,36^. Online research is also less well-controlled than laboratory research. However, numerous investigations have verified the validity and reliability of online studies, especially when using “catch trials” (as we used here) to filter out individuals who are not paying attention to the task demands^37^. Another limitation is that we did not control for potential knowledge of the correct answers to the scholastic facts or the trivia questions, thus participants could have known the answers to some of the questions. Likewise, we did not include a memory test of the trivia questions as in prior studies, thus we could not replicate prior findings of better memory for high versus low trivia question. It is also worth noting that there was no incentive (or “goal”) for processing the scholastic fact, as there would be in the domain of education where students would be incentivized to remember the scholastic fact, for example knowing that the material would be on a test. Finally, as noted above, the effects of memory encoding were tested immediately. While some studies have tested memory for trivia answer over a long-term retention interval^12,38^, the longest intervals used to test the spillover effect for incidental information has so far been only 24-h. In real-world academic situations, testing is very likely to occur days or even weeks after learning. Investigating how curiosity affects the long-term retention of incidental unrelated information is of high interest.

## Methods

### Participants

We recruited participants via the CloudResearch platform, an Amazon Mechanical Turk toolkit that generates “approved participants” through an extensive evaluation and appraisal of data quality through the assessment of participant attention, engagement, and English comprehension^39^. A total of 250 participants enrolled in one of three task versions described below. Eligibility was restricted to individuals aged 18–50, living in the United States, and who spoke English as their first language. After excluding datasets that contained >10 missing trials, and that failed >2/3 attention checks (*N* = 11), *N* = 239 (122 women [participants reported their gender], mean age = 33.9 [range 18–50]) remained for the main analysis. In this sample, 15.5% (*N* = 37) had advanced degrees (PhD, Master’s, or MD), 5.4% (*N* = 13) were in graduate school, 43.9% (*N* = 105) had a bachelor’s degree, 34.7% (*N* = 83) had a high school diploma, and 0.42% (*N* = 1) did not have a high school diploma. Study procedures were approved by the IRB at the University of Texas at Austin and all participants provided written informed consent.

### Trivia ratings

To ensure a range of low- and high-curiosity trivia facts, we generated a pool of 75 trivia questions of differing categories (animal trivia, celebrity trivia, and general knowledge trivia), e.g., “Q: How many inches of rain does Nebraska get annually? A: 23.6 inches of rain per year” vs. “Q: According to astronauts, what does the moon smell like? A: Burnt gunpowder”. These trivia questions were pilot rated by a separate pool of online participants (*N* = 100; 55 women, mean age = 33.1 [range 18–50]). Notably, this set of participants did not participate in the main experimental task. Participants rated each question from 1–4 based on the level of curiosity each question elicited, i.e., how curious they were in the answer to the question (1 = “not at all curious” and 4 = “very curious”). From these 75 trivia facts, we selected 20 questions with the lowest ratings (mean ± SEM: 2.11 ± 0.06) and 30 questions with the highest ratings (mean ± SEM: 3.26 ± 0.03) to use in our main task.

### Scholastic facts

Fifty neutral, scholastic facts were divided into English (*n* = 16) (e.g., Assonance is a literary term used to describe the repetition of similar vowel sounds in different words), history (*n* = 17) (e.g., Argentina joined Uruguay and Brazil against Paraguay in the War of the Triple Alliance., and science (*n* = 17) (e.g., Nonmetallic atoms attract electrons more strongly than metallic atoms) content. In general, the material was not common knowledge and resembled classroom material. Notably, these facts were unrelated to the trivia questions presented during the main task.

### Main task

The design for this study was adapted from (Gruber et al.,^11^) and was programmed and distributed on Qualtrics (Qualtrics, Provo, UT) (Fig. 1). There were three versions of this task. In the first two versions of the task, in line with Gruber et al.^11^, we were interested in exploring whether an anticipatory period during a state of curiosity—with varying anticipatory times—could enhance memory for incidental scholastic facts. In the third version of the task, we were interested in exploring whether the satisfaction generated by receiving the answer to a trivia question associated with high curiosity could also enhance memory for incidental scholastic facts. Before each task, participants were told that they would see a question, that they had to rate their curiosity about the answer from 1–4, and that they would also see the answer to that question. These questions and answers (trivia) were colored in green. Participants were also informed that they would see some facts that may not seem related to the trivia questions colored in green, but that it was still necessary to pay close attention to them, as we were recording their reaction times throughout the study. As this was only to get participants to pay attention to the scholastic fact, we did not specify any further details about reaction time recording. These facts (scholastic information) were colored in blue. Participants had three practice trials to orient them before the beginning of the study phase. Participants who completed one of the task versions were prevented from participating in the other two task versions, and participant assignment was quasi-random.

#### Version 1.1: Early anticipation period (*N* = 95, 48 women, mean age = 32.7 [range 20–50])

In this participant sample, 10.5% had an advanced degree, 7.4% were in graduate school, 44.2% had a bachelor’s degree, and 37.9% had a high school diploma. In this task version, a trial began with the presentation of a trivia question and a subsequent rating from 1–4, where participants had to rate how curious they were to receive the answer to the trivia question (1 = “not at all curious” and 4 = “very curious”). Immediately after, participants would see a scholastic fact, unrelated to the trivia question. The presentation of a scholastic fact was followed by the answer to the trivia question that was initially displayed. A 2-s crosshair followed each trial, and each stimulus (trivia question, scholastic fact, and trivia answer) lasted 4 s. In all three versions of the task, following 4 s, participants could press an arrow to continue, meaning they pressed an arrow following each trivia question, scholastic fact, and trivia answer (Fig. 1A).

#### Version 1.2: Late anticipation period (*N* = 48, 26 women, mean age = 35.7 [range 21–48])

In this participant sample, 25% had an advanced degree, 4.2% were in graduate school, 50% had a bachelor’s degree, and 20.8% had a high school diploma. In an attempt to mitigate interference from the initial trivia question and the scholastic fact, we created a task version with a longer anticipatory period between the presentation of the trivia question, and the scholastic fact. Here, everything else was kept the same as the Early Anticipation Task, except that the initial trivia rating was followed by the presentation of a 4-s crosshair before the presentation of the scholastic fact (Fig. 1B).

#### Version 2.1: Post satisfaction period (*N* = 96, 48 women, mean age = 34.1 [range 19–50])

In this participant sample, 15.6% had an advanced degree, 4.2% were in graduate school, 40.6% had a bachelor’s degree, 38.5% had a high school diploma, and 1.0% had not finished high school. In this task version, a trial began with the presentation of a trivia question and a subsequent rating from 1–4, where participants had to rate how curious they were to receive the answer to the trivia question. The trivia answer immediately followed the rating of the trivia question. Subsequently, participants would see the scholastic fact. This was the main difference between this version of the task and Early and Late Anticipation versions, as the scholastic fact was presented *following* the trivia answer instead of preceding the trivia answer. A 2-s crosshair followed each trial, and each stimulus (trivia question, scholastic fact, and trivia answer) had a duration of 4 s (Fig. 1C).

### Recognition memory test for incidental scholastic material

Following the end of the main task, there was a brief delay period where participants watched a neutral 1-min video of a boat in the water. Analogous to a pop quiz, we administered a 53-question surprise multiple-choice test on the scholastic facts presented during the main task. Each multiple-choice question had four choices, and participants were instructed to select the choice that best completed the fact they saw in the initial portion of the experiment (e.g., Assonance, a literary term, describes which of the following? (A) A word used to imitate a sound, (B) A repetition of similar words in a sentence, (C) An overuse of conjunctions in a sentence, or (D) A repetition of similar vowel sounds in different words.) Three of these 53 multiple-choice questions were catch trials to ensure participants’ attention throughout the recognition memory test (if they failed more than two or three of these catch trials, they were eliminated) (e.g., Please answer this question by choosing the second option, “a little bit”. (A) A lot, (B) A little bit, (C) Quite a bit, or (D) Quite a lot). Multiple-choice questions were presented in random order and varied per participant (i.e., the order of a question pertaining to a particular scholastic fact did not match the order it was presented in during the task).

### Data processing and analytic plan

All data processing and analyses were conducted in the R environment (R Core Team, 2018).

#### Pre-determined high vs. low curiosity ratings

To test whether pre-determined categories of low vs. high curiosity (determined by the separate pool of online participants) had an effect on incidental memory for scholastic facts, we used ANOVA fit with the ez package on R^40^ that included within-subjects factors of pre-determined curiosity (high vs. low) and between subjects factors of task version (early anticipation, late anticipation, and post satisfaction). Post hoc two-tailed paired t-tests followed main effects or interactions. Memory accuracy was assessed as each participant’s average of correct responses (during the multiple-choice test) for scholastic questions paired with low curiosity trivia vs. high curiosity trivia.

#### Individual curiosity ratings

In addition, we fit a generalized mixed effect model with a binomial outcome (memory accuracy) to analyze how individual ratings of curiosity affected memory for scholastic facts. Primary analyses were done within the mixed-effects regression framework; all models were fit using the *lme4* library^41^. In this model, fixed effects included trial-level curiosity ratings, mean curiosity rating for each participant (in order to account for between-subjects variance in average curiosity), trial number and finally, as a categorical fixed effect, task version (early anticipation, late anticipation, and post satisfaction). A random intercept for subjects was included. To assess the individual model terms for significance, we used Wald *z*-tests, per standard recommendations for GLMMs without overdispersion^42^. The fitted model showed no signs of overdispersion (dispersion ratio = 0.949, *p* = 1). We present unstandardized beta coefficient (*b*) in odds-ratio (OR) form to facilitate the interpretation of memory performance.

### Reporting summary

Further information on research design is available in the Nature Research Reporting Summary linked to this article.

### Supplementary information

reporting summary

## Data Availability

Data and stimulus materials are available on https://github.com/dunsmoorlab and the Open Science Framework.
