# Supplementary material for: States of epistemic curiosity interfere with memory for incidental scholastic facts
Source: NPJ Sci Learn. 2024 Mar 18;9:22. doi: 10.1038/s41539-024-00234-w (PMC10948872; doi:10.1038/s41539-024-00234-w)
Supplement: Supplementary file 1 — reporting summary [file 41539_2024_234_MOESM1_ESM.pdf]

Corresponding author(s): Joseph Dunsmoor

Last updated by author(s): Nov 17, 2023

Reporting Summary

Nature Portfolio wishes to improve the reproducibility of the work that we publish. This form provides structure for consistency and transparency in reporting. For further information on Nature Portfolio policies, see our Editorial Policies and the Editorial Policy Checklist.

Statistics

For all statistical analyses, confirm that the following items are present in the figure legend, table legend, main text, or Methods section.

n/a	Confirmed	
☐	☒	The exact sample size (n) for each experimental group/condition, given as a discrete number and unit of measurement
☐	☒	A statement on whether measurements were taken from distinct samples or whether the same sample was measured repeatedly
☐	☒	The statistical test(s) used AND whether they are one- or two-sided<br>*Only common tests should be described solely by name; describe more complex techniques in the Methods section.*
☐	☒	A description of all covariates tested
☐	☒	A description of any assumptions or corrections, such as tests of normality and adjustment for multiple comparisons
☐	☒	A full description of the statistical parameters including central tendency (e.g. means) or other basic estimates (e.g. regression coefficient) AND variation (e.g. standard deviation) or associated estimates of uncertainty (e.g. confidence intervals)
☒	☐	For null hypothesis testing, the test statistic (e.g. F, t, r) with confidence intervals, effect sizes, degrees of freedom and P value noted<br>*Give P values as exact values whenever suitable.*
☒	☐	For Bayesian analysis, information on the choice of priors and Markov chain Monte Carlo settings
☒	☐	For hierarchical and complex designs, identification of the appropriate level for tests and full reporting of outcomes
☐	☒	Estimates of effect sizes (e.g. Cohen's d, Pearson's r), indicating how they were calculated

Our web collection on statistics for biologists contains articles on many of the points above.

Software and code

Policy information about availability of computer code

Data collection	Programmed and distributed on Qualtrics (Qualtrics, Provo, UT).
Data analysis	We used R, as described in the text.

For manuscripts utilizing custom algorithms or software that are central to the research but not yet described in published literature, software must be made available to editors and reviewers. We strongly encourage code deposition in a community repository (e.g. GitHub). See the Nature Portfolio guidelines for submitting code & software for further information.

Data

Policy information about availability of data

All manuscripts must include a data availability statement. This statement should provide the following information, where applicable:

- Accession codes, unique identifiers, or web links for publicly available datasets
- A description of any restrictions on data availability
- For clinical datasets or third party data, please ensure that the statement adheres to our policy

Data and stimulus materials are available on the Open Science Framework: https://osf.io/u3z7x/?view_only=dc54943a21a740b9ad60d6ba17769364.

Research involving human participants, their data, or biological material

Policy information about studies with human participants or human data. See also policy information about sex, gender (identity/presentation), and sexual orientation and race, ethnicity and racism.

Reporting on sex and gender	Sex or gender was not considered in the study design. Subjects reported their "gender."
Reporting on race, ethnicity, or other socially relevant groupings	We do not report race. In this sample, 15.5% (N = 37) had advanced degrees (PhD, Master's, or MD), 5.4% (N = 13) were in graduate school, 43.9% (N = 105) had a bachelor's degree, 34.7% (N = 83) had a high school diploma, and 0.42% (N = 1) did not have a high school diploma.
Population characteristics	Eligibility was restricted to individuals aged 18-50, living in the United States, and who spoke English as their first language.
Recruitment	We recruited participants online, via the CloudResearch platform, an Amazon Mechanical Turk toolkit that generates "approved participants" through an extensive evaluation and appraisal of data quality through the assessment of participant attention, engagement, and English comprehension.
Ethics oversight	Study procedures were approved by the IRB at the University of Texas at Austin and all participants provided written informed consent.

Note that full information on the approval of the study protocol must also be provided in the manuscript.

Field-specific reporting

Please select the one below that is the best fit for your research. If you are not sure, read the appropriate sections before making your selection.

☐ Life sciences ☒ Behavioural & social sciences ☐ Ecological, evolutionary & environmental sciences

For a reference copy of the document with all sections, see nature.com/documents/nr-reporting-summary-flat.pdf

Behavioural & social sciences study design

All studies must disclose on these points even when the disclosure is negative.

Study description	Quantitative
Research sample	The sample size was N = 239 (122 women [participants reported their gender], mean age = 33.9 [range 18 - 50]). In this sample, 15.5% (N = 37) had advanced degrees (PhD, Master's, or MD), 5.4% (N = 13) were in graduate school, 43.9% (N = 105) had a bachelor's degree, 34.7% (N = 83) had a high school diploma, and 0.42% (N = 1) did not have a high school diploma
Sampling strategy	Random. The sample size was based on prior research using this task design, as detailed in the manuscript. We did not conduct a priori Power Calculations.
Data collection	We recruited participants via the CloudResearch platform, an Amazon Mechanical Turk toolkit. The study was conducted online using Amazon Mechanical Turk.
Timing	Version 1.1, Short anticipation period: 8/20/2021-9/8/2021<br>Version 1.2, Longer anticipation period: 3/18/2022-3/22/2022<br>Version 2.1, Satisfactory period: 12/10/2021-12/15/2021
Data exclusions	Excluded participant datasets that contained > 10 missing trials, and that failed > 2/3 attention checks (N = 11) during the online study.
Non-participation	No participants dropped out.
Randomization	Participants who completed one of the task versions were prevented from participating in the other two task versions, and participant assignment was quasi-random.

Reporting for specific materials, systems and methods

We require information from authors about some types of materials, experimental systems and methods used in many studies. Here, indicate whether each material, system or method listed is relevant to your study. If you are not sure if a list item applies to your research, read the appropriate section before selecting a response.

Materials & experimental systems

n/a	Involved in the study
☒	☐ Antibodies
☒	☐ Eukaryotic cell lines
☒	☐ Palaeontology and archaeology
☒	☐ Animals and other organisms
☒	☐ Clinical data
☒	☐ Dual use research of concern
☒	☐ Plants

Methods

n/a	Involved in the study
☒	☐ ChIP-seq
☒	☐ Flow cytometry
☒	☐ MRI-based neuroimaging

Plants

Seed stocks	*Report on the source of all seed stocks or other plant material used. If applicable, state the seed stock centre and catalogue number. If plant specimens were collected from the field, describe the collection location, date and sampling procedures.*
Novel plant genotypes	*Describe the methods by which all novel plant genotypes were produced. This includes those generated by transgenic approaches, gene editing, chemical/radiation-based mutagenesis and hybridization. For transgenic lines, describe the transformation method, the number of independent lines analyzed and the generation upon which experiments were performed. For gene-edited lines, describe the editor used, the endogenous sequence targeted for editing, the targeting guide RNA sequence (if applicable) and how the editor was applied.*
Authentication	*Describe any authentication procedures for each seed stock used or novel genotype generated. Describe any experiments used to assess the effect of a mutation and, where applicable, how potential secondary effects (e.g. second site T-DNA insertions, mosiacism, off-target gene editing) were examined.*
